# Supplementary material for: Long-lasting severe immune dysfunction in Ebola virus disease survivors
Source: Nat Commun. 2020 Jul 24;11:3730. doi: 10.1038/s41467-020-17489-7 (PMC7381622; doi:10.1038/s41467-020-17489-7)
Supplement: Supplementary file 1 — Supplementary Information [file 41467_2020_17489_MOESM1_ESM.pdf]

Supplementary Information

Long-lasting severe immune dysfunction in Ebola virus disease survivors

Wiedemann A. *et al.*

Postebogui Study Group

Ahidjo Ayouba [1], Sylvain Baize [2], Kaba Bangoura [3], Alimou Barry [1], Moumié Barry [3], Mamoudou Cissé [4], Mohammed Cissé [3], Eric Delaporte [1], Jean-François Delfraissy [5], Christelle Delmas [5], Alice Desclaux [1], Saliou Bella Diallo [3], Mamadou Safiatou Diallo [3], Mariama Sadjo Diallo [3], Jean François Étard [1], Cécile Etienne [5], Ousmane Faye [3], Ibrahima Fofana [6], Bruno Granouillac [1], Esther Hereth Hébert [1] , Suzanne Izard [1], Djenaba Kassé [3], Alpha Kabinet Keita [1], Sakoba Keita [7], Lamine Koivogui [8], Cécé Kpamou [1], Christine Lacabaratz [9], Sandrine Leroy [1], Claire Levy Marchal [10], Yves Levy [9], N'Fally Magassouba [11], Laura March [1], Vincent Mendiboure [12], Philippe Msellati [1], Harissatou Niane [3], Martine Peeters [1], Yves-Marie Pers [1], Hervé Raoul [13], Sidi Lamine Sacko [6], Ibrahima Savané [6], Mamadou Saliou Sow [3], Bernard Taverne [1], Abdoulaye Touré [14], Fodé Amara Traoré [3] , Falaye Traoré [8], Yamoussa Youla [15], Yazdan Yazdanpanah [5]

Affiliations

1. TransVIHMI, IRD UMI 233, INSERM U1175, Montpellier University, France
2. Pasteur Institute/Unit of Biology of Emerging Viral Infections, Lyon, France
3. Donka National Hospital, Conakry, Guinea
4. Forecariah Prefectoral Hospital, Forecariah, Guinea
5. Reacting, INSERM, Paris, France
6. Macenta Prefectoral Hospital, Macenta, Guinea
7. Ministry of Health, Conakry, Guinea
8. Institut National de la Santé Publique, Conakry, Guinea
9. Vaccine Research Institute, Université Paris-Est Créteil, Faculté de Médecine, INSERM U955, Team 16, Créteil, France
10. Pôle de Recherche Clinique (PRC), INSERM, Paris, France
11. Laboratory of Virology, Conakry University, Projet de Recherche sur les Fièvres Hémorragiques en Guinée, Conakry, Guinea
12. ALIMA, Conakry,Guinea
13. Laboratoire P4 Inserm-Jean Mérieux, US003 Inserm, Lyon, France
14. Department of Public Health, Conakry University, Conakry, Guinea
15. N'Zérékoré Regional Hospital, N'Zérékoré, Guinea

Supplementary Figures

Supplementary Figure 1: Clinical events and biological data of Ebola survivors enrolled in the Postebogui cohort, Guinea, 2014–16 [1]. Reprinted from The Lancet Infectious Diseases **17,** Etard, J.-F. *et al.* Multidisciplinary assessment of post-Ebola sequelae in Guinea (Postebogui): an observational cohort study. 545-552, 4855950138989 (2020) with permission from Elsevier

	All patients (n=802)	Age group		
		Adults (n=644)	Children (n=158)	p value
**Clinical events**				
Median days between start of symptoms and inclusion (IQR)	118 (10–321)	138 (15–337)	65 (5–259)	0·0002
All symptoms*	606 (76%)	505 (78%)	101 (64%)	0·0003
General symptoms	324 (40%)	250 (39%)	74 (47%)	0·07
Fever	209 (26%)	151 (23%)	58 (37%)	0·0011
Fatigue	190 (24%)	154 (24%)	36 (23%)	0·83
Anorexia	89 (11%)	57 (9%)	32 (20%)	0·0002
Abdominal symptoms	198 (25%)	163 (25%)	35 (22%)	0·47
Abdominal or pelvic pain	178 (22%)	143 (22%)	35 (22%)	1·0
Gastritis	56 (7%)	54 (8%)	2 (1%)	0·0007
Ocular symptoms and signs	142 (18%)	124 (19%)	18 (11%)	0·0200
Conjunctivitis	33 (4%)	27 (4%)	6 (4%)	1·0
Iridocyclitis	11 (1%)	9 (1%)	2 (1%)	1·0
Cataract	11 (1%)	10 (2%)	1 (1%)	0·70
Glaucoma	9 (1%)	8 (1%)	1 (1%)	1·0
Vision problems	101 (13%)	95 (15%)	6 (4%)	0·0001
Ocular pain	34 (4%)	25 (4%)	9 (6%)	0·38
Musculoskeletal symptoms	303 (38%)	274 (43%)	29 (18%)	<0·0001
Neck pain	10 (1%)	9 (1%)	1 (1%)	0·70
Back pain	56 (7%)	54 (8%)	2 (1%)	0·0007
Joint pain	254 (32%)	228 (35%)	26 (16%)	<0·0001
Myalgia	111 (14%)	99 (15%)	12 (8%)	0·0100
Neurosensory disorders	298 (37%)	231 (36%)	67 (42%)	0·14
Headache	278 (35%)	212 (33%)	66 (42%)	0·0402
Dizziness	19 (2%)	18 (3%)	1 (1%)	0·15
Other	20 (2%)	17 (3%)	3 (2%)	0·78
Auditory symptoms (deafness)	19 (2%)	16 (2%)	3 (2%)	1·0
**Biological characteristics**				
Haemoglobin				
Median (g/dL; IQR)	12·1 (10·9–13·8)	12·5 (11·0–14·0)	11·4 (10·3–12·5)	<0·0001
Anaemia (<11 g/dL)	186/724 (26%)	132/587 (22%)	54/137 (39%)	<0·0001
Severe anaemia (<8 g/dL)	12/724 (2%)	7/587 (1%)	5/137 (4%)	0·0575
Patient data missing	78 (10%)	57 (9%)	21 (13%)	
Creatinine value				
>100 µmol/L	60/583 (10%)	60/474 (13%)	0	<0·0001
>150 µmol/L	2/583 (<1%)	2/474 (<1%)	0	1·0
Patient data missing	219 (27%)	170 (26%)	49 (31%)	
Aspartate aminotransferase				
Aspartate aminotransferase >40 UI/L	52/431 (12%)	33/345 (10%)	19/86 (22%)	0·0027
Patient data missing	371 (46%)	299 (46%)	72 (46%)	
C-reactive protein				
C-reactive protein >10 mg/L	43/562 (8%)	35/461 (8%)	8/101 (8%)	0·84
C-reactive protein >40 mg/L	9/562 (2%)	7/461 (2%)	2/101 (2%)	0·67
Patient data missing	240 (30%)	183 (28%)	57 (36%)	

Data are n (%) unless stated otherwise. *All symptoms presented in the table.

Categorical data are presented as n (%) and continuous data as median (IQR). χ^2 test or Fisher exact tests were used for comparisons between adults and children.

Supplementary Figure 2: Distribution of clinical signs at the harvest time (V0), within one month (M1) and within three months (M3) of n=34 EBOV_S

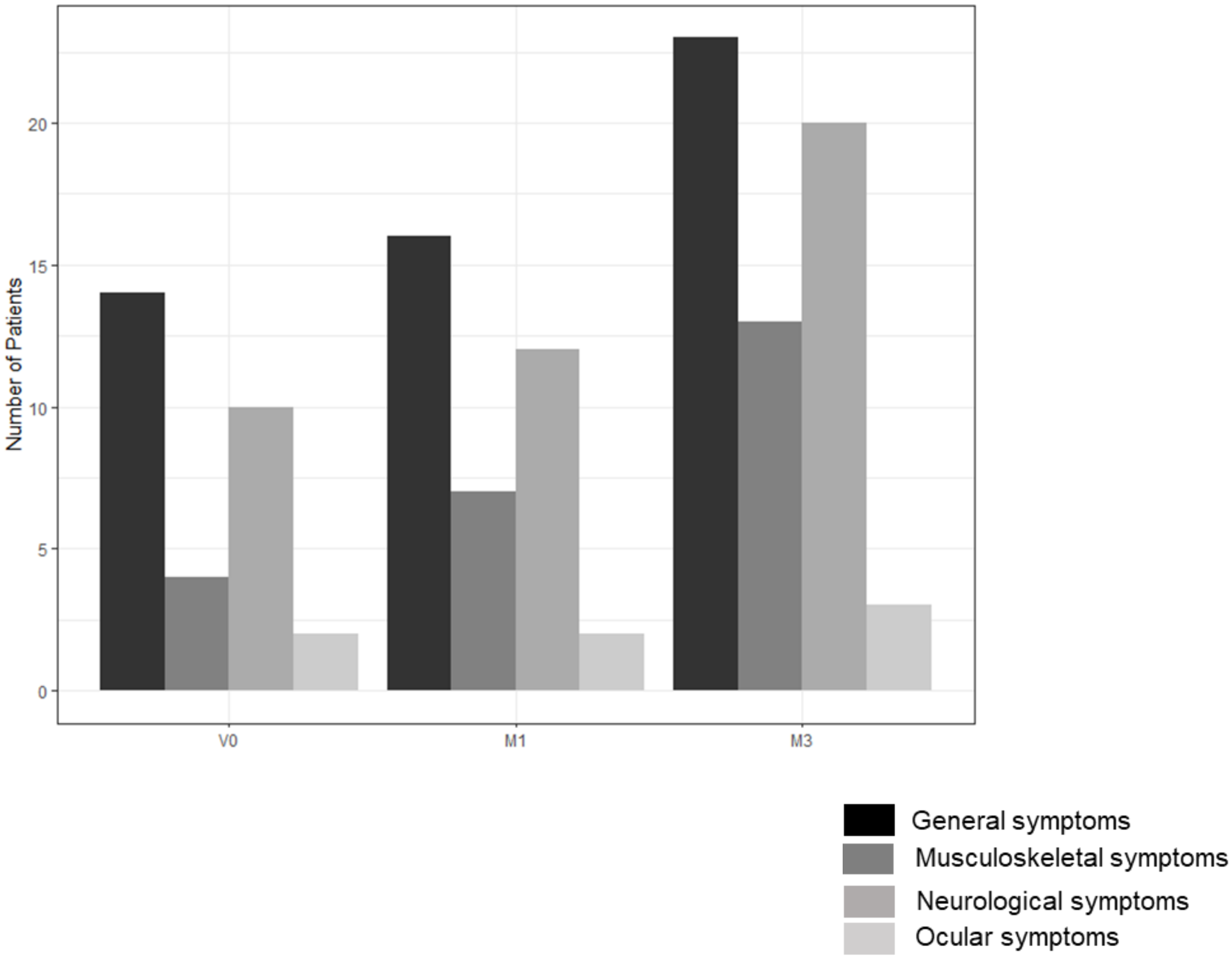

Symptoms were sub-divided in 4 main categories: general symptoms (fatigue, anorexia, fever, pallor, abdominal pain, and pelvic pain), musculoskeletal symptoms (arthralgia, myalgia), neurological symptoms (headache, insomnia, vertigo, sensory disorders), ocular symptoms (conjunctivitis, ocular disorders, ocular pain)

Supplementary Figure 3: Determination of EBOV antibodies with Luminex technology

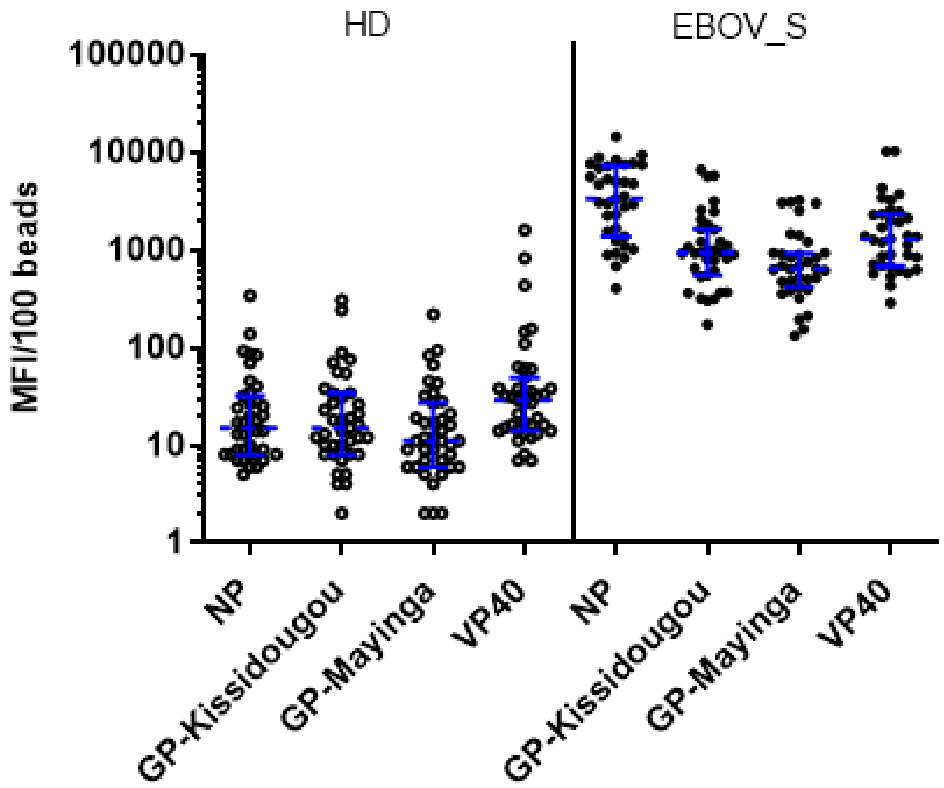

Serum samples from 39 HD (left) and plasma samples from 35 EBOV_S (right) were assessed for the presence of IgG against various recombinant Ebola virus proteins, as indicated. The positivity criteria were: nucleoprotein (NP): MFI≥600 and glycoprotein (GP): MFI≥400 (at least one of two GP) or positive for NP, viral protein (VP40) and 1 of 2 GP 200< MFI<399 (weak responder). Median values ± IQR are shown (blue). Source data are provided as a Source Data file.

Supplementary Figure 4: PBMC phenotypic characterization

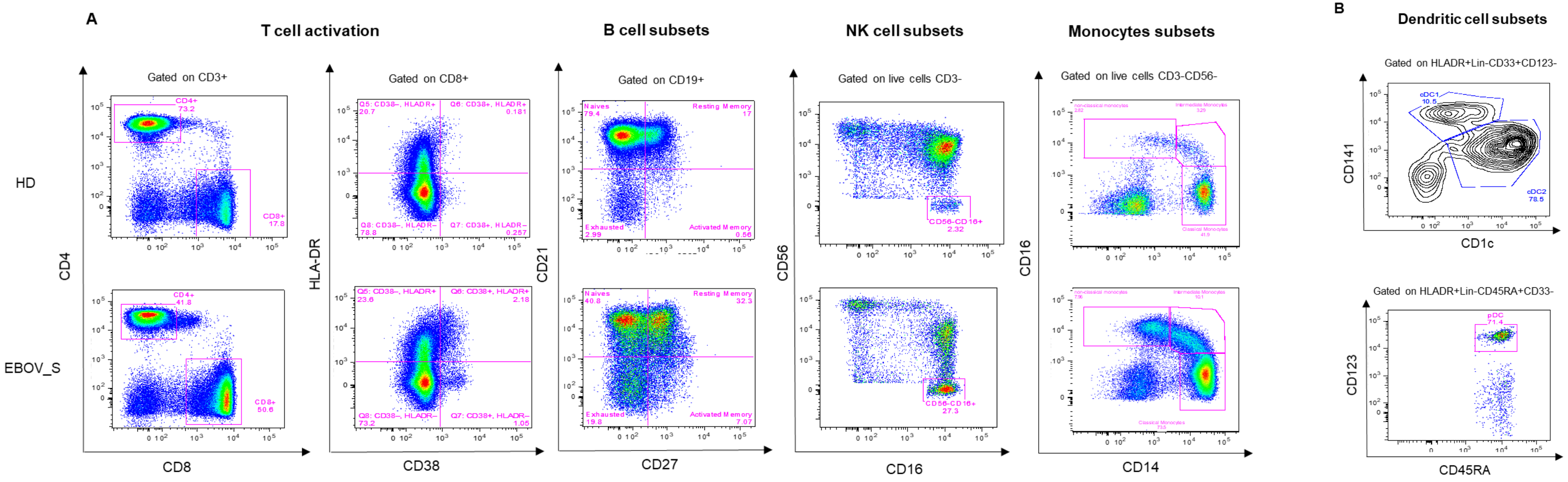

Representative flow cytometry results for the phenotypic characterization of PBMC from one HD (upper panel) and one EBOV_S (lower panel) (A). Gating strategy for the phenotypic characterization of DC (B).

Supplementary Figure 5: Cytokine profiles of PBMC from EBOV_S stimulated *in vitro* with EBOV peptides

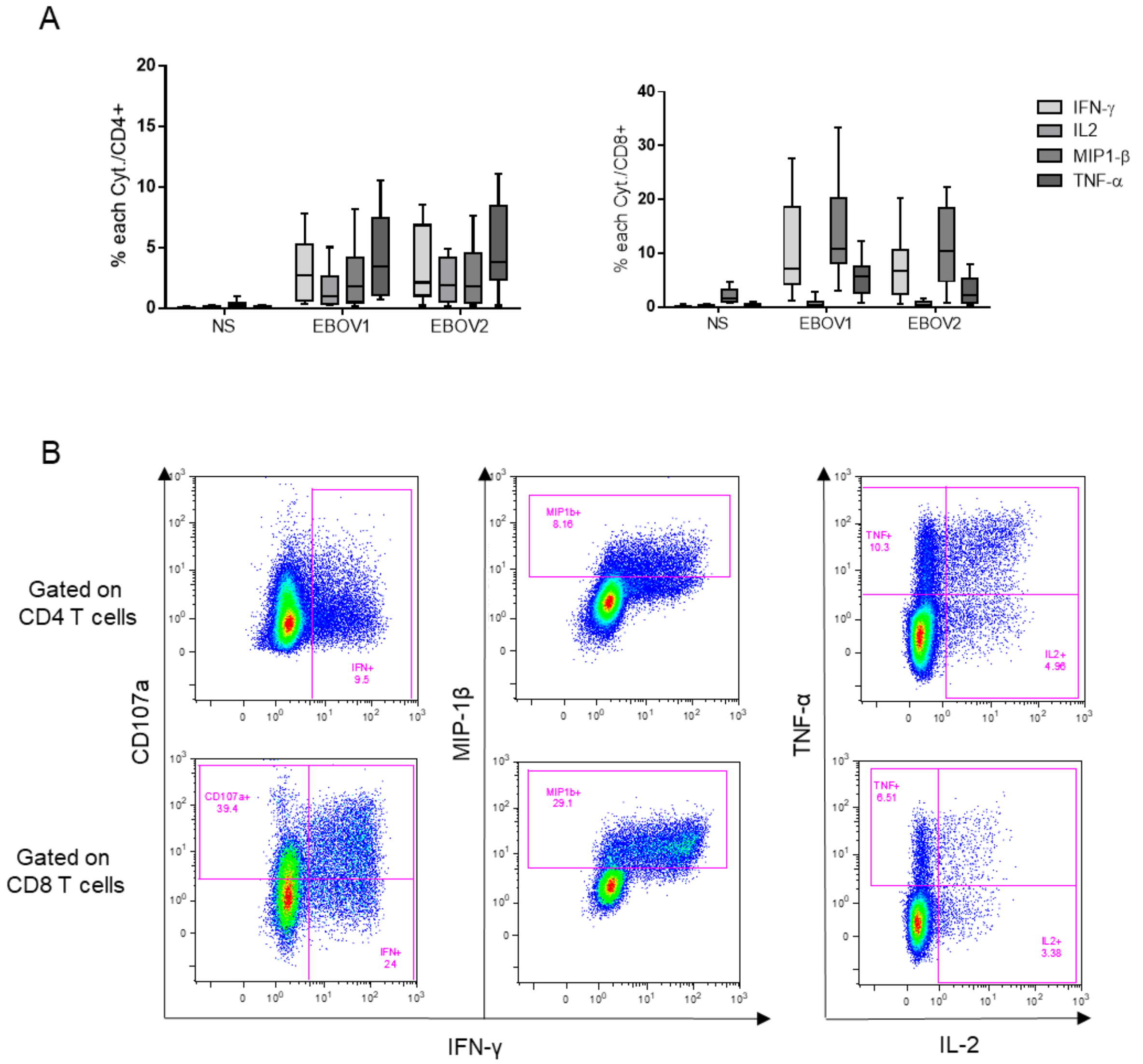

Frequency of CD4$^+$ T cells producing IFN-γ, IL2, MIP-1β and TNF after the stimulation of PBMC from EBOV_S (*n*= 27) with 2 EBOV-GP peptide pools (EBOV1 and EBOV2) for 9 days (left). *P*<0.0001 for each cytokine relative to non-stimulated cells (NS). Frequency of CD8$^+$ T cells producing IFN-γ, IL-2, MIP-1β and TNF-α after the stimulation of PBMC from EBOV_S (*n*=27) with 2 EBOV-GP peptide pools (EBOV1 and EBOV2) for 9 days (right). *P*<0.0001 for each cytokine except IL-2 (*P*=0.013 NS/EBOV1; no difference NS/EBOV2) (A). Representative dot plots of EBOV GP-specific CD4$^+$ and CD8$^+$ T-cell responses from EBOV_S after 9 days of EBOV GP-specific T-cell expansion *in vitro* (EBOV1 peptide pool) (B). Box plots represent cumulative data for EBOV_S. The lower and upper bounderies of the box represent the 25th and 75th percentiles, with a line indicating the median; whiskers represent the 10th and 90th percentiles. Friedman's test was used. Source data are provided as a Source Data file.

Supplementary Figure 6: Cytokine profiles of PBMC from HD stimulated *in vitro* with EBOV peptide

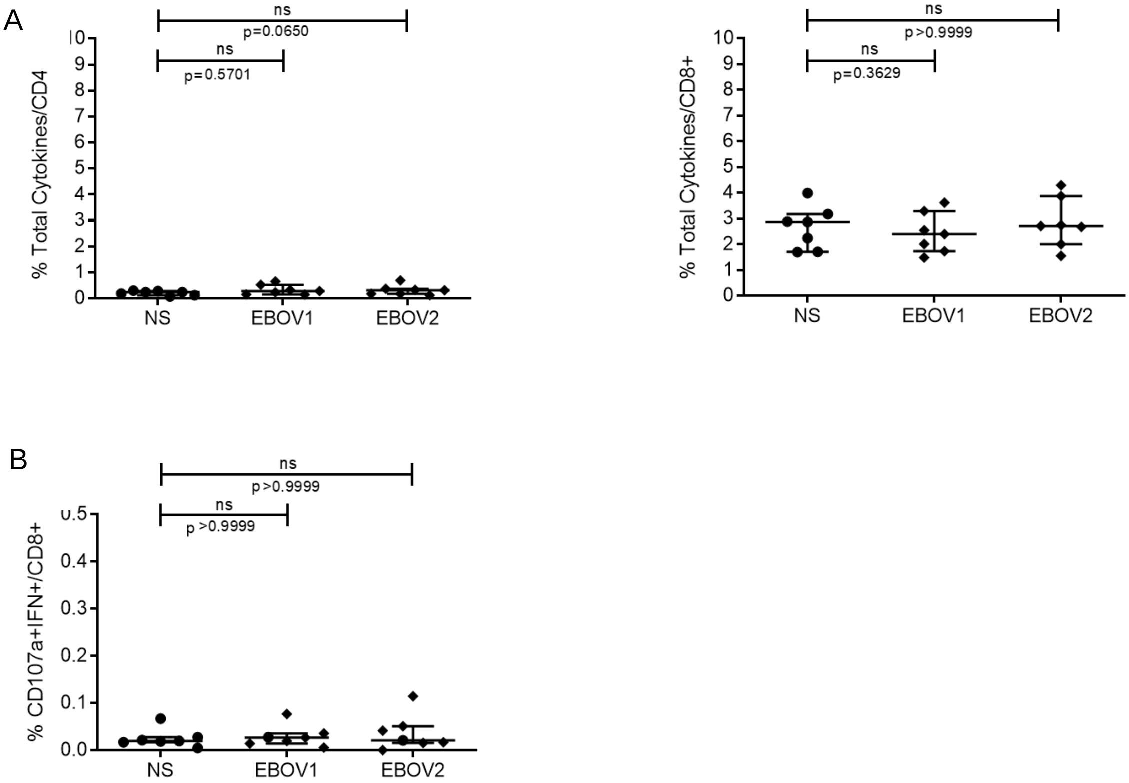

EBOV GP-specific CD4+ T-cell (left panel) and CD8+ T-cell (right panel) responses of HD (*n*=7) after nine days of EBOV GP-specific (EBOV1 and EBOV2 peptide pools) T-cell expansion *in vitro* (all cytokines) (A). Analysis of the co-expression of CD107a and IFN-γ by EBOV GP-specific CD8 T cells from HD (*n*=7) after nine days of antigen-specific T-cell expansion *in vitro* (B). Median values ± IQR are shown, and Friedman's test was used for comparisons. Source data are provided as a Source Data file.

Supplementary Figure 7: Distribution of immunological markers according to the presence of clinical symptoms

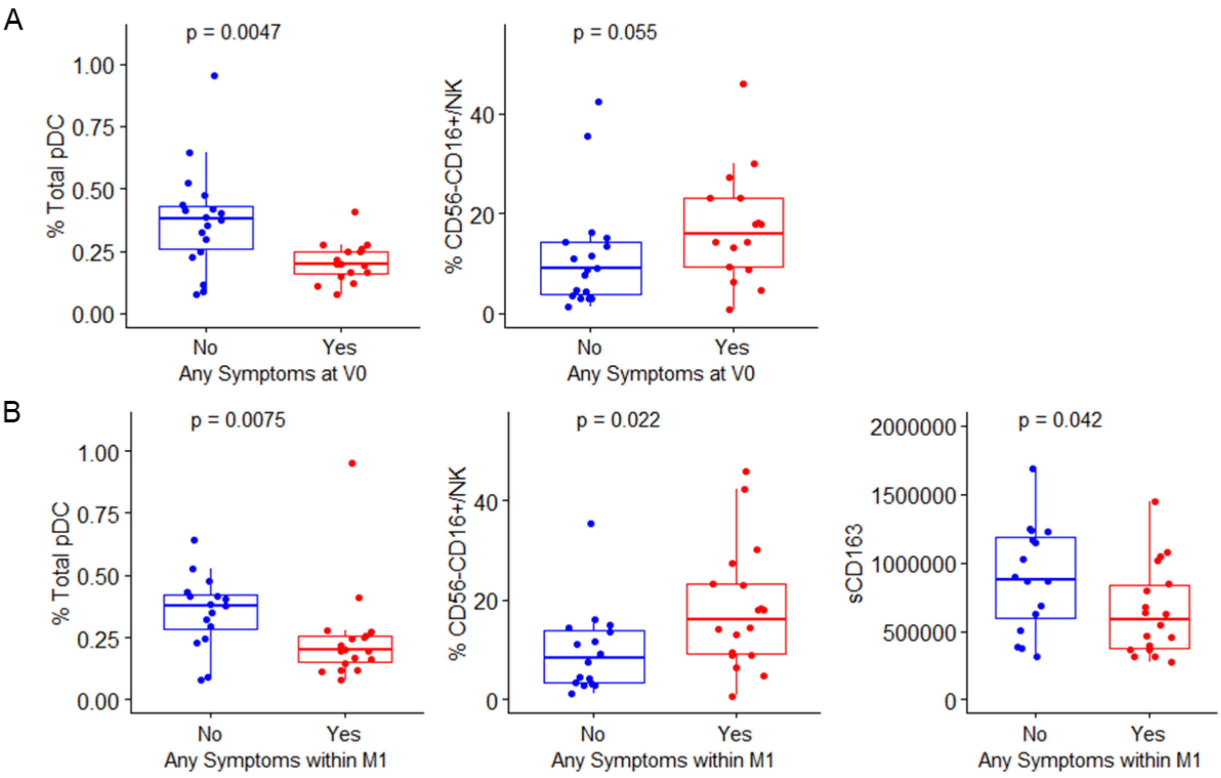

Frequency of total pDC and non-classical NK cells according to the presence (red) or absence (blue) of clinical signs at V0 (A). Frequency of total pDC and non-classical NK cells and measurement of seric sCD163 according to the presence (red) or absence (blue) of clinical signs within M1 after the harvest time (B).The differences between the two groups were evaluated using Wilcoxon rank sum statistical test. The lower and upper bounderies of the box represent the 25% and 75% percentiles, the whiskers extend to the most extreme data point which is no more than 1.5 times the interquartile range away from the box. Median values (horizontal line in the boxplot) are shown

Supplementary Figure 8 : List of Genes from acute EBOV transcriptomic signature [2] belonging to Chaussabel's "Interferon-inducible" M3.1 module [3]

LAP3, EIF2AK2, IFI35, OAS1, LGALS3BP, OAS3, OAS2, IFIH1, FBXO6, GBP1, IFIT3, GCH1, EPSTI1, RSAD2, OASL, IFI44L, IFI44, HERC6, HERC5, PML, CACNA1A, IFITM3, SERPING1, ANKRD22, MX1, LY6E, CXCL10, IRF7, PLSCR1, PGAP1

References

1 Etard, J.-F. *et al.* Multidisciplinary assessment of post-Ebola sequelae in Guinea (Postebogui): an observational cohort study. *The Lancet Infectious Diseases* **17**, 545-552, doi:10.1016/s1473-3099(16)30516-3 (2017).

2 Liu, X. *et al.* Transcriptomic signatures differentiate survival from fatal outcomes in humans infected with Ebola virus. *Genome Biol* **18**, 4, doi:10.1186/s13059-016-1137-3 (2017)

3 Chaussabel, D. *et al.* A modular analysis framework for blood genomics studies: application to systemic lupus erythematosus. *Immunity* **29**, 150-164, doi:10.1016/j.immuni.2008.05.012 (2008)
