# Supplementary material for: Long-lasting severe immune dysfunction in Ebola virus disease survivors
Source: Nat Commun. 2020 Jul 24;11:3730. doi: 10.1038/s41467-020-17489-7 (PMC7381622; doi:10.1038/s41467-020-17489-7)
Supplement: Supplementary file 3 — Reporting Summary [file 41467_2020_17489_MOESM3_ESM.pdf]

natureresearch

Corresponding author(s): Yves Lévy

Last updated by author(s): May 25, 2020

Reporting Summary

Nature Research wishes to improve the reproducibility of the work that we publish. This form provides structure for consistency and transparency in reporting. For further information on Nature Research policies, see Authors & Referees and the Editorial Policy Checklist .

Statistics

For all statistical analyses, confirm that the following items are present in the figure legend, table legend, main text, or Methods section.

n/a	Confirmed	
☐	☒	The exact sample size (*n*) for each experimental group/condition, given as a discrete number and unit of measurement
☐	☒	A statement on whether measurements were taken from distinct samples or whether the same sample was measured repeatedly
☐	☒	The statistical test(s) used AND whether they are one- or two-sided<br>*Only common tests should be described solely by name; describe more complex techniques in the Methods section.*
☒	☐	A description of all covariates tested
☐	☒	A description of any assumptions or corrections, such as tests of normality and adjustment for multiple comparisons
☐	☒	A full description of the statistical parameters including central tendency (e.g. means) or other basic estimates (e.g. regression coefficient) AND variation (e.g. standard deviation) or associated estimates of uncertainty (e.g. confidence intervals)
☐	☒	For null hypothesis testing, the test statistic (e.g. *F*, *t*, *r*) with confidence intervals, effect sizes, degrees of freedom and *P* value noted<br>*Give P values as exact values whenever suitable.*
☒	☐	For Bayesian analysis, information on the choice of priors and Markov chain Monte Carlo settings
☒	☐	For hierarchical and complex designs, identification of the appropriate level for tests and full reporting of outcomes
☐	☒	Estimates of effect sizes (e.g. Cohen's *d*, Pearson's *r*), indicating how they were calculated

Our web collection on statistics for biologists contains articles on many of the points above.

Software and code

Policy information about availability of computer code

Data collection	DIVA v6.2 (cytometer), Bio-Plex Manager v6.1 (Luminex), Hi-Seq Controler (Sequencing). FastQ files were generated from .bcl files on BaseSpace Sequence Hub 5.45. After trimming (QPhred score ≥ 25), reads were aligned to the hg19 human reference genome [https://www.ncbi.nlm.nih.gov/assembly/GCF_000001405.13/], using STAR - v. 2.5.3ar, and quantified relative to annotation model hg19 - GENCODE Genes - release 19 [https://www.gencodegenes.org/human/release_19.html], with Partek E/M Build version 9.0.20.0514
Data analysis	Sequencing quality control was performed with Sequence Analysis Viewer (SAV) version 2.1.8. Statistical analysis were performed with Prism v8 for Windows (Graphpad Software Inc)or with SAS (version 9.3 or higher, SAS Institute, Cary, NC, USA), R (version 3.6, The R Foundation for Statistical Computing, Vienna, Austria) and XLSTAT (version 2011.4.04, Addinsoft, Paris, France). Flow cytometry data were analyzed with FlowJo v9 (Treestar) and SPICE v5.22 (http://exon.niaid.nih.gov/spice). Gene enrichment analysis was analyzed with Ingenuity Pathway software v.51963813.

For manuscripts utilizing custom algorithms or software that are central to the research but not yet described in published literature, software must be made available to editors/reviewers. We strongly encourage code deposition in a community repository (e.g. GitHub). See the Nature Research guidelines for submitting code & software for further information.

Data

Policy information about availability of data

All manuscripts must include a data availability statement. This statement should provide the following information, where applicable:

- Accession codes, unique identifiers, or web links for publicly available datasets
- A list of figures that have associated raw data
- A description of any restrictions on data availability

RNA sequencing data that support the findings of this study have been deposited in Gene Expression Omnibus (GEO) repository with the accession codes GSE143549 [https://www.ncbi.nlm.nih.gov/geo/query/acc.cgi?acc=GSE143549]. The source data underlying Figs 1, 2, 3; Table 1 and Supplementary Figs 3,5 and 6 are provided as a Source Data File.

Field-specific reporting

Please select the one below that is the best fit for your research. If you are not sure, read the appropriate sections before making your selection.

☒ Life sciences ☐ Behavioural & social sciences ☐ Ecological, evolutionary & environmental sciences

For a reference copy of the document with all sections, see nature.com/documents/nr-reporting-summary-flat.pdf

Life sciences study design

All studies must disclose on these points even when the disclosure is negative.

Sample size	We enrolled a subgroup (n=35) of post-EVD survivors from the Postebogui cohort in this ancillary immunological study in Guinea. The design of the Postebogui cohort and patient characteristics have been described previously. Eligible patients (adults who accepted additional visits and blood samples) with laboratory-confirmed EVD subsequently declared virus-free were recruited at the ETCs in Guinea between March 2015 and July 2016. The healthy volunteers (n=39) were enrolled in the PREVAC (Partnership for Research on Ebola Vaccination) vaccine trial Guinean center agreeing to participate in the immunological evaluation were included, at baseline, as controls. Sample size was chosen to ensure similar group size between survivors and HD groups in order to maximize statistical power given the total number of observations
Data exclusions	All experiments were performed on frozen cells in Paris, France. Pre-established exclusion criteria were: after thawing, cells with viability <75% were not processed; for mRNA sequencing, samples with RNA Integrity Number <7 were not processed.
Replication	All experiments included a sufficient sample size, taking into account the expected variability when using human PBMC, serum and blood. Representative data were confirmed at least once with an independent experiment.
Randomization	This is not a randomized study: Individuals enrolled are survivors from EVD participating in a large survey cohort of Ebola survivors in Guinea. A control group of healthy individuals living in the same aera was included. Participants in the two group were randomly selected.
Blinding	Data collection/generation and analysis was not blinded to the operator for the different discovery experiments. Samples were collected from 2 different sites in Guinea and were differentially identified.

Reporting for specific materials, systems and methods

We require information from authors about some types of materials, experimental systems and methods used in many studies. Here, indicate whether each material, system or method listed is relevant to your study. If you are not sure if a list item applies to your research, read the appropriate section before selecting a response.

Materials & experimental systems

n/a	Involved in the study	
☐	☒	Antibodies
☒	☐	Eukaryotic cell lines
☒	☐	Palaeontology
☒	☐	Animals and other organisms
☐	☒	Human research participants
☐	☒	Clinical data

Methods

n/a	Involved in the study	
☒	☐	ChIP-seq
☐	☒	Flow cytometry
☒	☐	MRI-based neuroimaging

Antibodies

Antibodies used	Multiparametric flow cytometry panel was performed using a battery of antibodies : anti-CD38 FITC #340909, anti-HLADR PE #347401, anti-CD4 BV421 #562424 , anti-CD8 APCH7 #560179, anti-CD3 Alexa 700 #557943, anti-CCR7 Alexa647 #557734, CD21 PE #555422, CD27 APC #337169, CD45 Alexa 700 #560566, anti-CD56 PECF594 #564849, anti-HLADR BV605 #562845, anti-CD33 BV421 #562854, anti-CD141 BV711 #563155, anti-CD45RA PerCpCy5.5 #563429, anti-HLA ABC BV786 #740982, anti-CD86 PECF594 #562390 (all from BD Biosciences), anti-CD45RA PEefluor 610 #61-0458-42(ebiosciences); anti-CD19 PC7 #IM3628 (Beckman Coulter), anti-CD38 PercpCy5.5 #303522, anti-IgM Pacific Blue #314514, anti-CD71 BV650 #334116, anti-CD20 APCCy7 #302314, anti-CD16 APC Cy7 #302018, anti-CD14 BV605 #301834, anti-CD161 BV421 #339914, anti-NKG2D PercPcy5.5 #320818, anti-NKp46 PC7 #331916, anti-CD1c PECy7 #331516, anti-CD40 PE #334308, Lineage FITC #348801(Biolegend), IgD FITC #H15501 (Invitrogen), anti-CD123 APC #130-113-322(Miltenyi Biotec). For ICS analyses, cells were stained with surface monoclonal antibodies: anti-CD4 PE PECF594 # 562281, anti-IFNγ FITC #557718, anti-TNFα PE-Cy7 #557647 , anti-MIP1β PE #550078 and anti-IL2 BV421 #564164 (all from BD Biosciences)
Validation	All antibodies were commercially available (Reactivity: Human (QC testing), Application: flow cytometry (routinely tested). See the corresponding manufacturer datasheets on webpages for reference and validation

Human research participants

Policy information about studies involving human research participants

Population characteristics	We enrolled 35 EBOV survivors, with a median age of 30 years (interquartile range (IQR): 25-36) (54% male) and 39 healthy donors (median age of 25 years [21-36] (80% male). Median [IQR] time between ETC discharge and enrollment was 23 months [19-25]. No EBOV RNA was detectable in the blood at time of sampling. The enrolled subjects received only supportive care (no experimental drugs, no convalescent plasma) during the acute phase of EBOV infection and were seronegative for HIV, HCV and HBV. On inclusion in Postebogui cohort, 23 of the 35 patients (66%) had post-EVD symptoms similar to those for the whole cohort . EBOV-specific antibodies was against NP, GP-Kissidougou, GP-Mayinga, and VP40,were detected in all survivors. By contrast, none of the serum samples from HD tested positive for these antibodies (Fig. 2, supplementary appendix).
Recruitment	EVD survivors were recruited from the Postebogui cohort . Eligible patients with laboratory-confirmed EVD subsequently declared virus-free were recruited at the ETCs in Guinea . All patients gave immunological study-specific written informed consent. Healthy volunteers enrolled in the PREVAC (Partnership for Research on Ebola Vaccination) vaccine trial Guinean center agreeing to participate in the immunological evaluation were included, at baseline, as controls. Gender was not a selection criteria but due to the period of recruitment in the PREVAC vaccine trial, healthy donors were predominantly men as compared to EBOV_S (P=0.03).
Ethics oversight	The study protocols were approved by the Research Committee of the National Ebola Response Coordination and the National Ethics and Health Research Committee in Guinea and ethics committees in France (INSERM/CEEI, IRD/CCDE)

Note that full information on the approval of the study protocol must also be provided in the manuscript.

Clinical data

Policy information about clinical studies

All manuscripts should comply with the ICMJE guidelines for publication of clinical research and a completed CONSORT checklist must be included with all submissions.

Clinical trial registration	INSERM Protocol C15-03; ID-RCB : 2016-A01143-48 [https://ansm.sante.fr/Services/Obtenir-un-numero-d-enregistrement-pour-une-RIPH]
Study protocol	POSTEBOGUI Protocol: n°CEEI: 15-201, CCTIRS: 15.551, CNIL:915134
Data collection	Samples were collected in Conakry, Guinea between March 2015 and July 2016
Outcomes	To analyze inflammatory, phenotypic, functional and gene expression profiles of EVD survivors

Flow Cytometry

Plots

Confirm that:

- [x] The axis labels state the marker and fluorochrome used (e.g. CD4-FITC).
- [x] The axis scales are clearly visible. Include numbers along axes only for bottom left plot of group (a 'group' is an analysis of identical markers).
- [x] All plots are contour plots with outliers or pseudocolor plots.
- [x] A numerical value for number of cells or percentage (with statistics) is provided.

Methodology

Sample preparation	Peripheral Mononuclear cells (PBMC), serum and whole blood were frozen in Guinea.Cryopreserved PBMC were thawed and rested, fixed, permeabilized and stained in France according to the demands on each experiment. All details are mentioned in the Methods section
Instrument	LSRII Fortessa 4-laser (488, 640, 561 and 405 nm) cytometer (BD Biosciences)
Software	Data were collected on DIVA v6.2 and analyzed using FlowJo software version 9.9.6 (Tree Star inc.)
Cell population abundance	1-2M of cells were stained and collected on cytometer.
Gating strategy	Gating strategies are described in supplementary figures 3 and 4

- [x] Tick this box to confirm that a figure exemplifying the gating strategy is provided in the Supplementary Information.
